# BMP4 and LGL1 are Down Regulated in an Ovine Model of Congenital Diaphragmatic Hernia

**DOI:** 10.3389/fsurg.2014.00044

**Published:** 2014-11-14

**Authors:** Heather M. A. Emmerton-Coughlin, K. Kathryn Martin, Jacky S. S. Chiu, Lin Zhao, Leslie A. Scott, Timothy R. H. Regnault, Andreana Bütter

**Affiliations:** ^1^Division of General Surgery, London Health Sciences Centre, The University of Western Ontario, London, ON, Canada; ^2^Department of Obstetrics and Gynaecology, London Health Sciences Centre, The University of Western Ontario, London, ON, Canada; ^3^Division of Pediatric Surgery, Children’s Hospital, The University of Western Ontario, London, ON, Canada; ^4^Children’s Health Research Institute, The University of Western Ontario, London, ON, Canada; ^5^Lawson Health Research Institute, The University of Western Ontario, London, ON, Canada

**Keywords:** congenital diaphragmatic hernia, pulmonary hypoplasia, sheep, Wnt, BMP4, LGL1

## Abstract

**Background/Purpose:** The molecular pathophysiology of lung hypoplasia in congenital diaphragmatic hernia (CDH) remains poorly understood. The Wnt signaling pathway and downstream targets, such as bone morphogenetic proteins (BMP) 4 and other factors such as late gestation lung protein 1 (LGL1), are essential to normal lung development. Nitrofen-induced hypoplastic CDH rodent lungs demonstrate down regulation of the Wnt pathway including BMP4 and reduced LGL1 expression. The aim of the current study was to examine the molecular pathophysiology associated with a surgically induced CDH in an ovine model.

**Methods:** Left thoracotomy was performed at 80 days in 14 fetal sheep; CDH was created in seven experimental animals. Lungs were harvested at 136 days (term = 145 days). Lung weight (LW) and mean terminal bronchiole density (MTBD) were measured to determine the degree of pulmonary hypoplasia. Quantitative real time PCR was undertaken to analyze Wnt2, Wnt7b, BMP4, and LGL1 mRNA expression.

**Results:** Total LW was decreased while MTBD was increased in the CDH group (*p* < 0.05), confirming pulmonary hypoplasia. BMP4 and LGL1 mRNA was significantly reduced in CDH lungs (*p* < 0.05). Wnt2 mRNA was decreased, although not significantly (*p* < 0.06).

**Conclusion:** For the first time, down regulation of BMP4 and LGL1 are reported in an ovine CDH model. In contrast to other animal models, these changes are persistent to near term. These findings suggest that mechanical compression from herniated viscera may play a more important role in causing pulmonary hypoplasia in CDH, rather than a primary defect in lung organogenesis.

## Introduction

Congenital diaphragmatic hernia (CDH) is a common congenital defect that occurs with a frequency of 1 in 2000 to 1 in 4000 live births (1). Despite prenatal diagnosis, advanced neonatal resuscitation, and intensive care, the mortality rate from CDH remains >20% due to severe pulmonary hypoplasia, pulmonary hypertension, and respiratory failure (2). The pathophysiology of CDH associated pulmonary hypoplasia remains poorly understood. Some authors postulate that the mechanical effect of the abdominal organs in the thoracic cavity may not fully account for the pulmonary hypoplasia found in CDH (3). In the nitrofen rodent model of CDH, early pulmonary hypoplasia occurs prior to diaphragmatic closure (3, 4). This hypoplasia is later exacerbated by the herniating abdominal viscera (3, 4). This dual-hit hypothesis postulates that diaphragmatic closure requires normally developing lungs (3). Nitrofen’s teratogenic effects on developing lungs as well as other organ systems further complicate this CDH model. In contrast to nitrofen, the ovine surgical CDH model is especially useful for molecular studies because it isolates the mechanical effect of CDH on lung development without affecting other organs. The fundamental pathogenesis of CDH in humans, whether due to failure of diaphragmatic closure disrupting lung development or due to a primary defect in lung organogenesis, remains unclear.

The Wnt signaling pathway plays a critical role in lung development. Numerous components within this pathway control cellular proliferation, differentiation, and lineage specification, as well as branching morphogenesis (5). Based on differences in the signal transduction pathway, Wnts can be categorized into two groups: canonical and non-canonical. Specifically, canonical Wnts, such as Wnt 2, modulate lung development early in organogenesis, while non-canonical Wnts, such as Wnt5a, regulate mid to late pulmonary development (5, 6). The persistent activation of the canonical Wnt pathway facilitates the development of alveolar epithelium in early embryonic lung endoderm, while it inhibits development of bronchiolar type epithelium (7). Embryos lacking Wnt2/2b experience complete agenesis of the lungs, while other foregut endoderm-derived organs are preserved (8). Inactivation of Wnt7b, a modulator of lung epithelial–mesenchymal interactions, causes decreased airway branching, pulmonary hypoplasia, and decreased lung smooth muscle (9, 10). Wnt7b null mutant mice die of respiratory failure at birth (9). In addition to the non-canonical Wnts, critical growth factors such as bone morphogenetic proteins (BMPs) have also been shown to contribute to lung organogenesis and development. BMP4, in particular, is a downstream target of the Wnt pathway and regulates proximal-distal patterning in addition to playing a role in modulating the lungs’ surfactant gene expression properties (11, 12). In the later stages of lung development, late gestation lung protein 1 (LGL1) plays a crucial role, orchestrating the regulation of alveolarization. It is a glycoprotein found in lung mesenchyme and has been shown to facilitate alveolarization and branching morphogenesis in fetal rat lung (13–15). LGL1 appears to be up regulated after reversal of pulmonary hypoplasia with tracheal occlusion in rats (16), highlighting deficiencies in LGL1 may impact normal alveologenesis.

Given the importance of the Wnt signaling pathway and its downstream targets, such as BMP4 and others factors such as LGL1, in lung development in the nitrofen CDH rat model, we sought to investigate if these molecular markers of lung growth were also down regulated in an ovine CDH model.

## Materials and Methods

### Animal model

Ethics approval for all animal experiments was obtained from Western University Animal User Subcommittee. Twelve time dated pregnant ewes were obtained and fasted 24 h prior to surgery. A left sided CDH was surgically created in seven fetal lambs at 80 days gestation as previously described (17). A sham left thoracotomy was performed in seven control lambs. Warmed normal saline along with Penicillin 1,000,000 IU was infused into the amniotic cavity prior to uterine closure. Each ewe received Trivetrim 1 ml/15 kg IM daily for the first three postoperative days.

At 129 days gestation, ewes received 250 mg medroxyprogesterone IM to prevent preterm labor (18). At 135 days gestation, all ewes received 0.5 mg/kg betamethasone IM since the fetuses were delivered prematurely (19). At 136 days gestation (term = 145 days), the fetuses were delivered by cesarean section after induction of general anesthesia of the ewe. Both the fetus and ewe were euthanized with an overdose of IV thiopental. A midline sternotomy exposed the fetuses’ thoracic contents. The presence, size, and position of the diaphragmatic hernia were recorded along with the type and amount of viscera herniating through the defect. The trachea was dissected and the heart and lungs removed en bloc. Total LW, followed by right and left LW, were measured after cutting the right and6 left main bronchi. Total, right, and left LW/body weight (LW/BW) were calculated.

Two of our five CDH sheep had premature closure of their diaphragmatic defect and were therefore excluded from analysis. Despite a standardized surgical protocol (20), these two sheep did not have any herniated viscera into their chest at delivery. Despite bringing two stomachs into the chest, early *in utero* reduction must have occurred, enabling the diaphragmatic defect to close.

### Tissue processing and analysis

Samples of the left lung were excised and rinsed in sterile PBS, frozen in liquid nitrogen, and stored at −80°C until RNA isolation. Additional pieces of the left and right lung were sectioned into 3–5 mm sections and were fixed in 4% paraformaldehyde and embedded in paraffin and stored at −20°C. Paraffin embedded samples were later sectioned (3–5 mm) and mean terminal bronchiole density (MTBD) for each lung was determined as previously described (21).

Total cellular RNA was isolated from the control and CDH left lung using TRIZOL reagent (Life Technologies, Railey, UK) according to the product protocol. RNA concentration was determined by standard spectrophotometric techniques and the RNA integrity was assessed by visual inspection of ethidium bromide-stained denaturing agarose gels. First-strand cDNA synthesis was carried out using SuperScript II Reverse Transcriptase System (Life Technologies, Inc., Carlsbad, CA, USA), as previously described (22). qRT-PCR was performed in triplicate on a BioRad CFX384 (BioRad, CA, USA), in mixtures of 12.5 μL Quanti Tect SYBR green (Qiagen, UK), 300 nM (each) primer, and 5 μL of diluted template DNA in a total volume of 25 μL. Primer sequences for Wnt2, Wnt7b, BMP4, and LGL1 are detailed in Table 1. Signal detection and analysis of results were performed with BioRad CFX384 sequence detection software (BioRad). Fold differences, normalized with 18s ribosomal RNA, were determined using the comparative ΔΔ CT method.

**Table 1:** **Primer sequences used for real time PCR**.

Gene		Sequence (5′-3′)
Wnt2	Forward	GAGGAAGTACAACGGGGCCA
	Reverse	TGTCCATGCCTCGGGAAGTC
Wnt7b	Forward	AGGCGCCTCATGAACCTTCA
	Reverse	CTTGATGCGCAGGAAGGTGG
BMP4	Forward	AGAGCCATGAGCTCCTTCGG
	Reverse	TCGTGGTGGAAGCTCCTCAC
Lgl1	Forward	ATGCTTCACAACAAGCTGC
	Reverse	GCTGGATGGACACTCAGAGC

We chose the following specific molecular markers in the Wnt pathway, Wnt2, Wnt7b, and BMP4, due to recent studies in the nitrofen-induced CDH rat model showing downregulation of these specific factors (23, 24). Similarly, LGL1, a marker of late lung development, was also shown to be downregulated in CDH rats but upregulated after tracheal occlusion (16).

### Statistical analysis

All data were reported as mean ± SD and were analyzed using Mann–Whitney *U* test, with statistical significance considered to be *p* < 0.05.

## Results

Of the 12 ewes, 2 were carrying twin gestations, which resulted in a total of 14 fetal lambs. CDH was created in seven fetuses (including the first in each twin gestation). Five singleton control fetuses and two control twins underwent sham thoracotomy. Two CDH fetuses and one singleton control aborted. Of the remaining five CDH lambs, two CDHs closed with no persistent diaphragmatic defect at delivery, leaving three CDH lambs. One singleton control delivered prematurely spontaneously and was excluded from analysis, leaving five control lambs (two control twins and three singletons).

**Figure 1:**
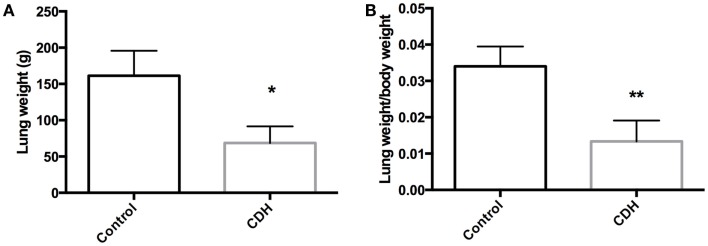
**(A)** Total lung weight and **(B)** lung to body weight ratio in CDH (*n* = 3) and control (*n* = 5) lambs following delivery at 136 days gestation. CDH lung weight **(A)** and lung to body weight ratio **(B)** were both significantly lower in the CDH sheep (*p < 0.05, **p < 0.01).

**Figure 2:**
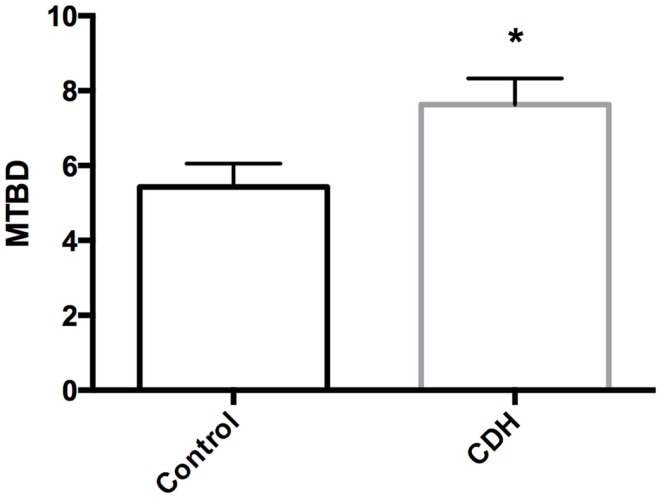
**Mean terminal bronchiole density in CDH lungs and controls**. MTBD was significantly higher in control (*n* = 5) compared with CDH (*n* = 3) lungs (*p < 0.05).

**Figure 3:**
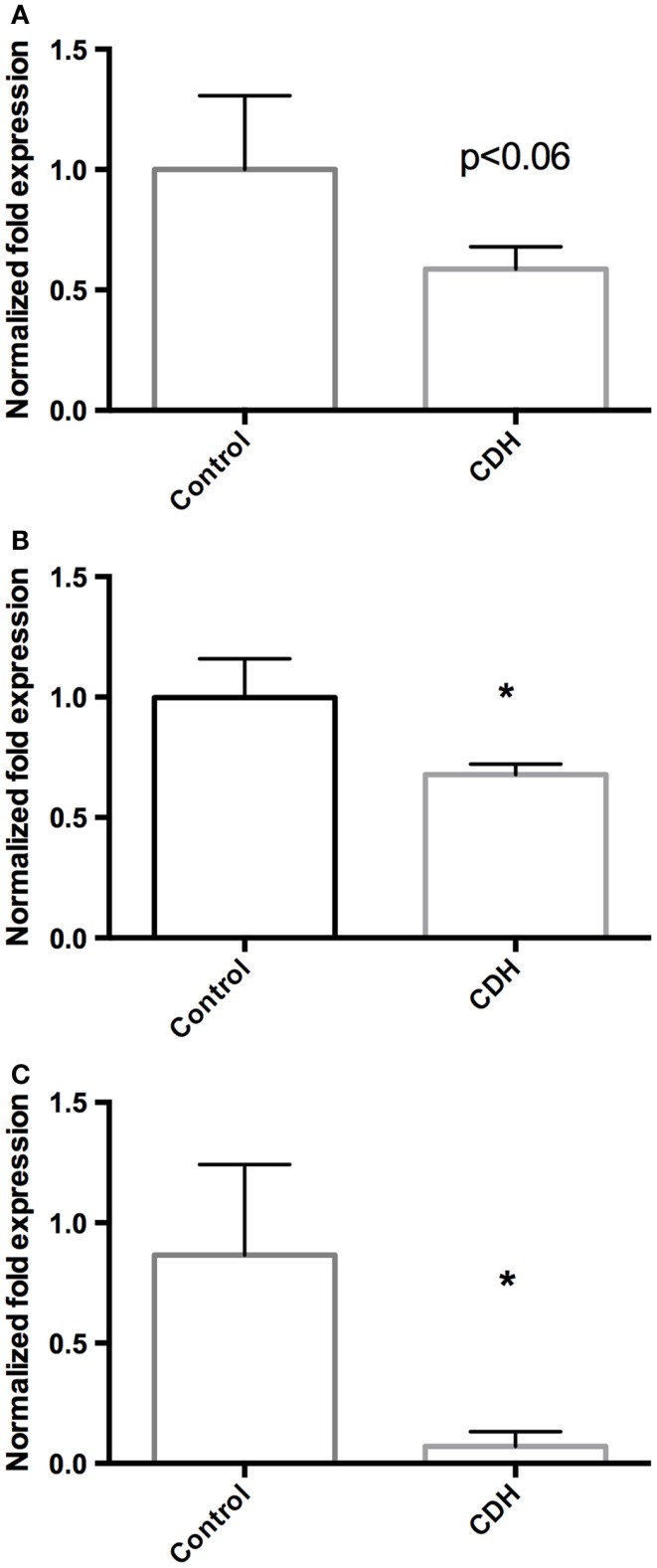
**(A)** Wnt2 mRNA expression normalized to 18S. Wnt2 expression was decreased in CDH lungs (*n* = 3) compared with controls (*n* = 5, *p* < 0.06), **(B)** BMP4 mRNA expression normalized to 18S. BMP4 expression was significantly decreased in CDH lungs (*n* = 3) compared with controls (*n* = 5, *p < 0.05), **(C)** LGL1 mRNA expression normalized to 18S. LGL1 expression was significantly decreased in CDH lungs (*n* = 3) compared with controls (*n* = 5, *p < 0.05).

Total LW (Figure 1A) and LW/BW ratio (Figure 1B) were found to be significantly decreased in the CDH lungs (*p* < 0.05, *p* < 0.01). MTBD was higher in CDH lungs (Figure 2, *p* < 0.05). Wnt2 was reduced in CDH lungs, albeit not significantly (Figure 3A, *p* < 0.06) and Wnt7b unchanged (data not shown). A downstream target of the Wnt signaling pathway, BMP4, was significantly decreased (Figure 3B, *p* < 0.05). In addition, a key regulator of late lung development, LGL1, was also significantly reduced in the lungs of CDH lambs (Figure 3C, *p* < 0.05).

## Discussion

Using an ovine CDH model, in conjunction with pulmonary hypoplasia, we report a reduced expression of Wnt2, BMP4, and LGL1, key players in promoting proximal-distal patterning, branching morphogenesis, and regulating alveolization (24–26). The confirmation of a reduced LW/BW ratios as well as increased MTBD highlighted pulmonary hypoplasia in our model. These term findings interestingly, correspond with the nitrofen-induced CDH rodent model induced changes observed between mid gestation and term (6, 22–26). Wnt2 is expressed at high levels in fetal rat lung mesenchyme (22), and in Wnt2 deficient mice, 50% die shortly after birth, presumably due to respiratory failure (23). In nitrofen treated animals to induce CDH, Takayasu et al. reported that Wnt2 and BMP4 were all significantly decreased in nitrofen treated animals compared with controls at E15, though Wnt2 and Wnt7b were not different from control near term at E21. They suggested that nitrofen causes down regulation of the Wnt signaling pathway, disrupting early lung development, and leading to pulmonary hypoplasia (24). They proposed a hierarchical model in which down regulation of Wnt genes causes decreased BMP4, resulting in diminished branching morphogenesis and pulmonary hypoplasia (24). Our study was not designed to examine the ontological changes in expression of these factors. However, it is interesting to note although Wnt2 was reduced in our CDH sheep (albeit not significantly), downstream BMP4, a key regulator of pulmonary development was significantly reduced near term, a similar finding to nitrofen treated animals (26).

LGL1, an essential gene in late lung development and alveolarization, and heterozygous LGL1 knockout mice exhibit disordered late lung development with areas of histologically immature lung characterized by thickened interstitial tissue (27). LGL1 was significantly decreased in our CDH lambs, a similar finding to the nitrofen treated CDH rats, where LGL1 was also reduced near term (E21) (25). Underlying mechanisms for this suppression remains unknown. Studies highlight that retinoic acid plays a critical role in modulating LGL1 during alveologenesis (25). Retinoic acid treatment in nitrofen-treated CDH rats results in up regulation of LGL1 (25). Similarly, tracheal occlusion also results in increased LGL1 in CDH rats (1, 16). Tracheal occlusion remains a controversial but potentially life-saving prenatal intervention in select CDH babies as it can reverse their pulmonary hypoplasia (1, 28). Therefore, LGL1 may provide a molecular basis to support the role of tracheal occlusion in mitigating the pathophysiologic effects of CDH.

Our study has some limitations. Firstly, given that this was a pilot study, we have small numbers of sheep. Secondly, immunohistochemistry (IHC) was not conducted and this would have strengthened our observations. Irrespective of these limitations, the near term mRNA changes occurring in concert with proven pulmonary hypoplasia (as evidenced by decreased lung wt/body wt and increased MTBD), a hallmark of CDH, make for an important observation and highlight further work should be conducted to fully understand the relationship between mRNA, protein, and the final phenotype in this surgical CDH model.

In conclusion, our findings provide a glimpse into the molecular signature of pulmonary hypoplasia in an ovine CDH model. We have shown that molecular markers of both early (BMP4) and late (LGL1) lung development are down regulated, in association with pulmonary hypoplasia at term. Unlike the nitrofen model of CDH, our ovine model does not involve the use of a potential teratogen on lung development (28). Therefore, it appears that the mechanical compression of herniated viscera on the developing lungs results in the persistent down regulation of BMP4 and LGL1, which results in pulmonary hypoplasia. These findings further support the idea that the persistent diaphragmatic defect *in utero* allowing visceral herniation is the issue, rather than a primary lung pathology causing pulmonary hypoplasia (3). Further studies into the Wnt pathways of sheep lungs, and eventually human lungs, are required to increase our understanding of the molecular pathophysiology of CDH and help guide future treatment for this condition.

## Conflict of Interest Statement

The authors declare that the research was conducted in the absence of any commercial or financial relationships that could be construed as a potential conflict of interest.
